# Functional outcome of surgical treatment of adults with extremity osteosarcoma after megaprosthetic reconstruction—single-center experience

**DOI:** 10.1186/s13018-019-1379-3

**Published:** 2019-11-07

**Authors:** Tomasz Goryń, Andrzej Pieńkowski, Bartłomiej Szostakowski, Marcin Zdzienicki, Iwona Ługowska, Piotr Rutkowski

**Affiliations:** Department of Soft Tissue/Bone Sarcoma and Melanoma, Maria Sklodowska-Curie Institute – Oncology Center, Warsaw, Poland

**Keywords:** Osteosarcoma, Combined treatment, Limb reconstruction

## Abstract

**Background:**

Osteosarcoma is the most common primary malignant bone tumor in adults and is usually located in the long bones. Standard treatment consists of perioperative chemotherapy and radical surgical resection. Limb-sparing surgery using a variety of reconstructive techniques remains the gold standard.

**Methods:**

In our study, we retrospectively analyzed 90 adult patients operated at our institution between 2000 and 2017 for extremity osteosarcoma that underwent limb-sparing reconstruction with the megaprosthesis. Sixty-one patients underwent resection and reconstruction of the distal femur, 9 patients—proximal femur, 7 patients—proximal tibia, 5 patients—total femoral resection and reconstruction, 5 patients—proximal humeral resection, and 3 patients—other types of resection with endoprosthetic reconstruction. The median follow-up time was 41 months, median overall survival was 86 months (3–225 months), and progression-free survival was 81 months (1–86 months). Functional assessment was made on 48 out of 56 living patients, after endoprosthetic reconstruction. The assessment was made according to MSTS functional scale.

**Results:**

In 14 cases (15%), the endoprosthesis had to be explanted, or amputation was performed for local recurrence or septic complication. Due to a mechanical failure of the implant, we had to perform a revision in 5 patients (5%). Eighteen out of 74 patients with endoprosthesis died of the disease. The median MSTS score was 84% (53–100%), and the best result of 85% was achieved in patients after distal femoral resection with endoprosthetic reconstruction.

**Conclusion:**

Careful planning of the treatment of patients with extremity osteosarcoma that is performed at the referral centers gives the possibility of long-term survival with a good and excellent functional result.

## Background

Osteosarcoma is the most commonly diagnosed primary malignant bone tumor with bimodal age distribution with its peak attributed to puberty and the fifth decade of life. The incidence of osteosarcoma in population is 0.2 per 100.000, which is less than 1% of solid tumors [1, 2]. Osteosarcoma commonly occurs in the long bones of the extremities. The most common sites include two of the bones forming the knee joint, followed by the proximal femur and the proximal humerus. Due to its rarity, osteosarcoma in adults remains a big challenge for determining a proper diagnosis and for setting correct treatment in a short period of time. The best treatment results are obtained with the use of combined therapy that includes perioperative chemotherapy, followed by radical surgical resection. At present, limb-sparing surgery (LSS) can be performed in the majority of patients with extremity osteosarcoma. Currently, various reconstructive techniques are used to restore the functional limb after extensive bone resection. The most frequently used are endoprosthetic joint reconstructions, arthrodesis implants, autografts, allografts, and custom-made implants.

Despite the increased risk of local recurrence, LSS is currently considered the optimal treatment solution. Published research shows that patients undergoing such treatment are characterized by a better total survival rate as compared with patients after amputation, not to mention reducing physical disability and its psychological effects that occur in patients undergoing such mutilating procedure [3–5].

Amputation is necessary only in 10–30% of patients with extensive involvement of surrounding soft tissues, vessels, or nerves, in poor general condition or after improper, earlier surgical treatment. It is caused mostly by delay in setting a proper diagnosis and treatment, which leads to more advanced disease at the time of diagnosis.

Due to more extensive muscle and bone resection in osteosarcoma patients, their functional results are inferior as compared to patients treated for degenerative disease.

Most papers which present the functional outcome of sarcoma patients after endoprosthetic reconstruction contain small non-homogenous groups of patients with a variety of diagnoses from metastatic lesions to the whole spectrum of primary bone tumors.

Many of them present the results of patients in a wide range of ages, from children to older people. In the case of children, their functional results are usually better than adults due to their better adaptation and regeneration possibility, which helps in more effective rehabilitation.

In most cases, the diagnosis in children is made earlier, and resection is not as extensive as in adults. The most worldwide adopted/approved scale to measure the functional outcome of patients after limb-sparing surgery is Musculoskeletal Tumor Society (MSTS) scoring scale. This scale can answer the question of how the patients benefit from a limb-sparing surgery and allow comparing the variety of reconstruction techniques.

The Department of Soft Tissue/Bone Sarcoma and Melanoma in one of the few referral centers in the East and Central Europe dedicated to complex treatment of the whole spectrum of bone sarcomas. In our department, patients are treated according to standard procedures adopted from NCCN and ESMO recommendation.

This study aimed to evaluate the functional results of adult patients treated for limb osteosarcoma in our referral center with megaprosthetic reconstruction.

## Material and methods

We had retrospectively analyzed the history of adult patients treated at our institution due to osteosarcoma in years 2000–2017 with the use of inclusion and exclusions criteria that are shown on Table 1.

**Table 1:** Study group inclusion and exclusion criteria

Inclusion criteria	Exclusion criteria
• Age > 18 years old	• Lost of follow-up
• Confirmed osteosarcoma in biopsy	• Surgery outside our department
• Extremity location	• Non-extremity location
• Limb-sparing surgery with the use of megaprothesis in our department	• Mutilating surgery
• Complete follow-up	

Finally, we have qualified 90 patients that underwent LSS with megaprosthetic reconstruction.

The indications for LSS in our group of patients were resectable osteosarcoma without neurovascular involvement and adequate soft tissue envelope for prosthetic coverage.

Tumor resectability was assessed on MRI or CT imaging performed after the third course of preoperative chemotherapy. According to AJCC classification in patients in stage IV of the disease, additionally, all metastases must be resectable after the preoperative chemotherapy courses. The resection of metastases was arranged after completing perioperative chemotherapy courses.

The chemotherapy was started after pathological diagnosis, and staging of osteosarcoma was confirmed in open or needle core biopsy of the tumor.

Routinely, we administered three courses of preoperative chemotherapy based on cisplatin and doxorubicin. From 2017 in patients under 30 years of age, we added methotrexate to routine chemotherapy. After surgery, patients received between 3 and 6 courses of chemotherapy according to the same preoperative schedule.

All patients were operated at our department by an experienced team of surgeons between 3rd and 4th weeks after the last course of neoadjuvant chemotherapy.

All patients underwent resection of osteosarcoma followed by reconstruction with megaprothesis.

In the first 18 patients treated with endoprosthetic reconstruction, we used custom-made implants. In the rest of patients, we implanted modular endoprosthetic tumor replacements (MUTARS by Implantcast GMbH Germany).

The minimum follow-up was 12 months from the end of treatment; the median follow-up time was 41 months. The mean age in the whole group of patients was 34 years (18–68). Patient characteristics are presented in Table 2.

**Table 2:** Type of reconstruction, the presence of pathological fracture, and the presence of metastases (M1) at the diagnosis in analyzed group of patients

Patient number	Distal femur	Proximal femur	Femur shaft	Proximal tibia	Proximal humerus	Others
Resection and reconstruction	61	9	5	7	5	3
M1	4	0	1	0	0	0
Pathological fracture	3	3	1	0	1	3

In Table 3, the stage of osteosarcoma according to AJCC staging classification version 8 in the analyzed group of patients is shown.

**Table 3:** Stage of osteosarcoma according to AJCC classification v.8 in analyzed group of patients

AJCC	Patient number
IA	4
IB	9
IIA	33
IIB	39
III	0
IV	5

All patients were followed-up in our outpatient clinic. At the time of their appointment, we assessed them against the following criteria: pain, function, acceptance of treatment, support, walking ability, and gait. Each evaluated category was rated from 0 to 6 points.

Functional effect of treatment was assessed in alive patients with implanted endoprosthesis with the help of the MSTS score system: 35 patients (88% alive patients) after distal femur resection and reconstruction, 4 patients (100% alive patients) after proximal femur resection and reconstruction, 2 patients (100% alive) after total femur resection, 5 patients (100% alive patients) after proximal tibia resection, and 2 patients (100% alive) after proximal humerus resection and reconstruction.

## Results

Median overall survival (OS) in the whole group of patients was 86 months (range 3–225 months), and progression-free survival (PFS) was 81 months (range 1–86 months).

During follow-up, 18 out of 74 patients died from the disease between 3 and 69 months after the surgery.

Age, survival time in months, AJCC stage of patients, who died, is shown in Table 4.

**Table 4:** Age, survival time, and stage of osteosarcoma according to AJCC classification of dead patients

Patients	Age	Survival time in months	AJCC stage
1	29	69	IIA
2	21	54	IIA
3	39	52	IIA
4	30	48	IIB
5	59	48	IIB
6	58	47	IV
7	43	43	IIB
8	23	28	IIB
9	19	32	IIB
10	30	22	IIA
11	37	21	IIA
12	20	21	IIB
13	56	21	IIB
14	26	14	IIB
15	30	13	IV
16	34	11	IIB
17	67	9	IV
18	22	3	IV

From a group of 90 patients after endoprosthetic reconstruction in 14 cases (15%), the endoprosthesis was explanted or the patient was amputated for local recurrence or septic complication.

We removed 9 of 61 (16%) distal femoral endoprosthesis, 3 of 5 (60%) proximal humerus, 1 (11%) proximal femur, and 1 of 7 (14%) proximal tibia endoprosthesis. Five patients (5%) had a revision for mechanical failure of the implant. During follow-up, 18 of 74 patients with endoprosthesis died of the disease.

**Fig. 1:**
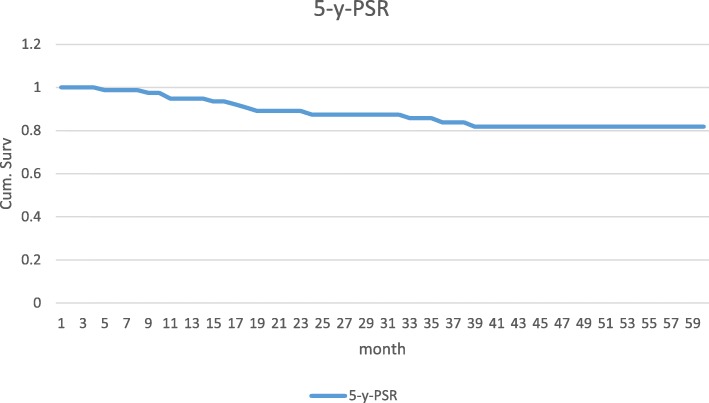
5-year prosthesis survival risk (5-y-PSR)

The prosthesis survival curve is presented in Fig. 1.

**Fig. 2:**
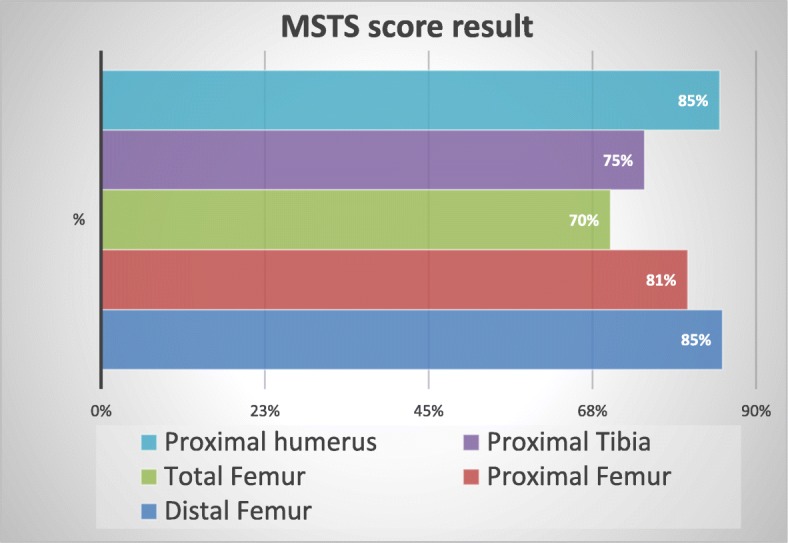
Total MSTS score results depending on resection

**Fig. 3:**
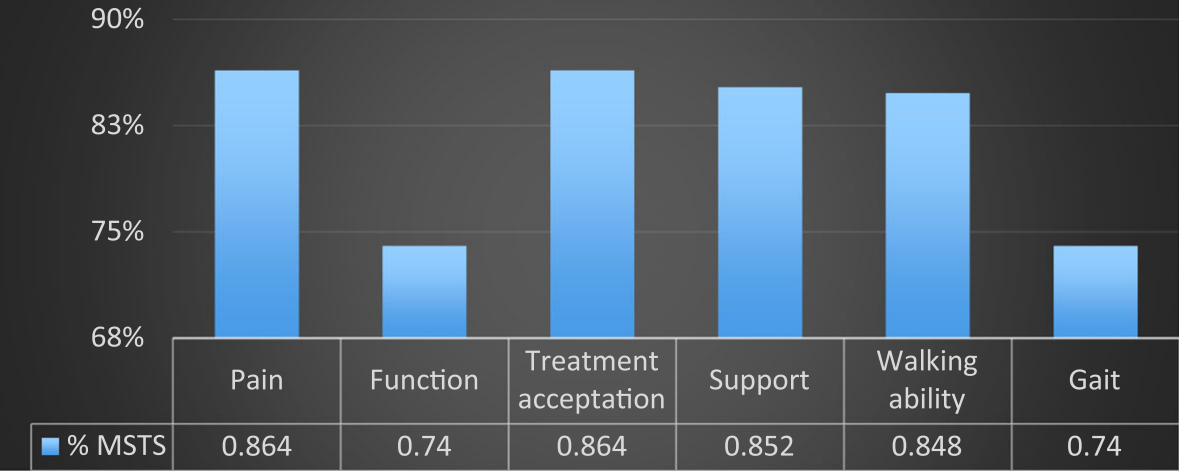
MSTS score results according to category

The results of MSTS evaluation are displayed in Figs. 2 and 3.

Twenty-four patients (49%) of all assessed patients achieved MSTS score better than 90% in the whole group and 20 patients (52%) in the group of patients after distal femur resection and reconstruction.

Achieved results show that patients had an excellent functional outcome of treatment, and they could return to a normal life after completing very demanding treatment.

**Fig. 4:**
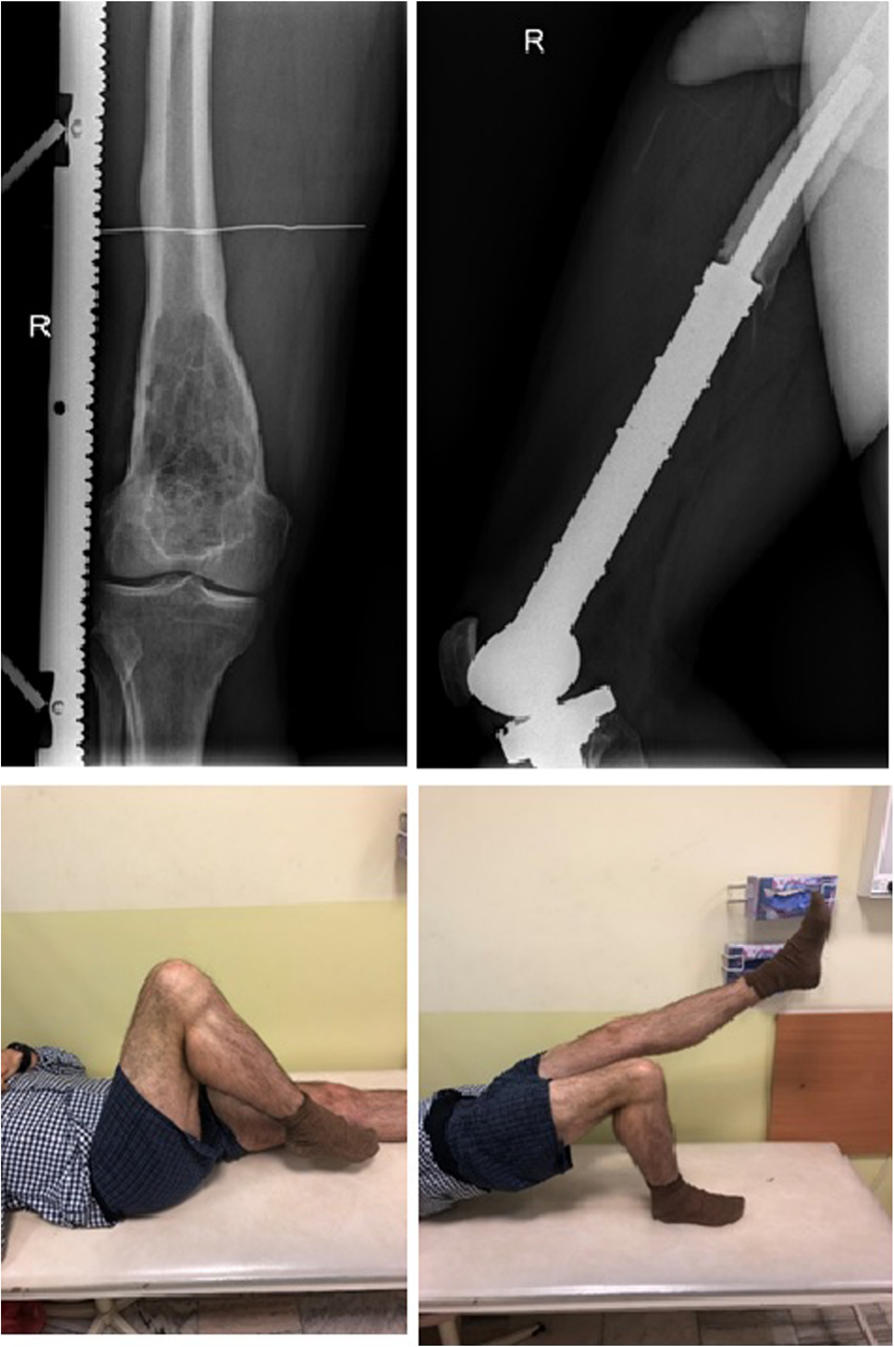
Results after resection and reconstruction of 20 cm of the right distal femur

The example of the reconstruction and functional results in a patient with osteosarcoma of the distal femur is shown in Fig. 4.

**Fig. 5:**
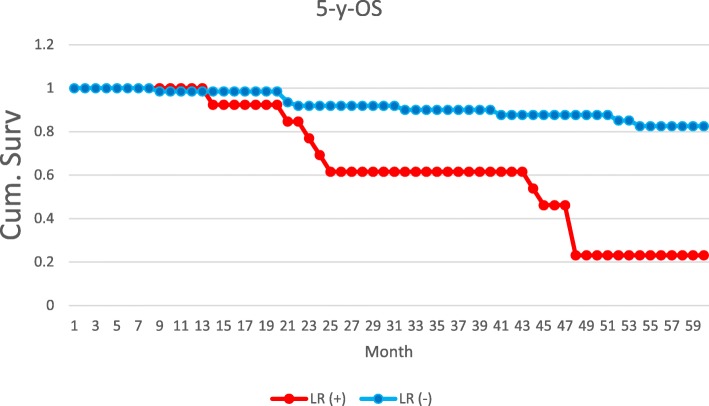
5-y-OS depending on presence of local reccurence /LR/

In our series, local recurrence (LR) occurred in 15 patients (16%), and its presence was related to poorer OS. In a group of patients with LR, metastases occurred in 13 patients (86%) and 13 patients (86%) died of the disease. Comparing to a group of 75 patients without LR who had much better results, in which metastases occurred only in 18 patients (24%) and 14 (18%) of them died of the disease (Fig. 5).

## Discussion

Department of Soft Tissue, Bone Sarcoma and Melanoma is a Polish referral center for the treatment of adult patients with the variety of primary bone and soft tissue sarcomas.

The treatment results in this department are similar to the results obtained in other reference centers.

Our treatment protocols are in line with recommended by ESMO, NCCN, and Polish Society of Oncology.

In our group of patients in I–III stages of the disease treated with resection and reconstruction, the 5-year OS rate is 74%.

That results are similar to the results presented by other European centers [6–9]. Results presented by German-Austrian-Swiss group in 1709 patients were slightly worst (5-year OS rate 49%), but the group also included patients treated with amputations [10].

Local recurrence (LR) occurred in our series in 16 patients (17.7%), and its presence was related to poorer OS. Occurring LR is connected to more aggressive disease and a much worse survival rate. LR is observed much more often in patients after non-radical resection (R1). LR occurred in 50% of patients with non-radical (R1) resection comparing with only 8.5% LR in patients after R0 resection.

The main goal of LSS is to avoid disability and to give the patient a chance to return to normal life after demanding and challenging treatment. In the lower limb, the minimal functional effect is to give the patient a chance to mobilize with support with preserved limb sensation.

In the upper limb, the minimal functional effect is hand grasping ability with preserved sensation. When this minimum requirement cannot be achieved with reconstructive surgery, it should be considered to perform amputation with an external prosthetic device. This type of treatment could give patients a better comfort of life than preserving the non-functional limb.

Functional results in patients treated at our department with megaprosthetic reconstruction can be considered good or very good.

The best results were achieved in patients after resection and reconstruction of tumor located in the distal femur with an average MSTS score of 85%.

More than half of patients from this group achieved MSTS score of 90% or higher, which means that they returned to normal life without disability seen.

In the majority of patients, resection and proper reconstruction allowed them to return to work or even to play recreational sports. That aspect of treatment is crucial to patients, especially at young age, to whom working or playing sport is an essential component of life quality. The worst functional results were obtained in patients after total femoral and proximal tibial reconstruction. These poor results could be caused by a small number of patients who were evaluated with the tumor in this particular location.

The other reason for poor results could be the fact that resection of a tumor located in the proximal tibia is connected with the necessity of resection and reconstruction of a patellar ligament in the proximal tibia resection and resection of muscles stabilizing the hip joint and pelvis in total femoral resection. Above can lead to delayed rehabilitation and irreversible disability.

In the majority of patients, reconstruction in the upper extremity can preserve the hand and elbow function, which helps to maintain an acceptable quality of life.

## Conclusion

Well planned and performed combined treatment of adult patients with extremity osteosarcoma leads to long-term survival with good or excellent functional results in the majority of patients.

The correct treatment is the most crucial, non-disease-related, and modified-able risk factor for obtaining patients with long-term survival.

Limb-sparing surgery is a gold standard of surgical treatment, and long-term survival rates are favorable in that group of patients comparing to a group of patients after amputation.

Majority of patients treated with resection and megaprosthetic reconstruction allowed them to return to normal life and work. It also reduces physical and mental disability.

Obtaining the correct and quick pathological diagnosis as well as starting the correct treatment in the referral center with no delay is the key to achieving the best results in patients with osteosarcoma.

Establishing accurate and early diagnosis could lead to a decreasing number of performing amputations and allowed to start treatment in patients with less advanced disease.

It is highly recommended to establish the referral center network for treating patients with sarcomas, including bone sarcomas, to give optimal diagnosis and treatment procedures without delay and to reduce the cost of treatment. That referral units participating in international clinical trials allow patients to get the latest and experimental therapies. Our department is a member of European Reference Network (ERN) for rare adult solid cancers (EURACAN), which gives our patient the best treatment opportunities.

## Data Availability

The dataset supporting the conclusions of this article is available on request—please contact the corresponding author MD Tomasz Goryń. Administrative permission was received from Maria Sklodowska-Curie Institute - Oncology Center, Warsaw, Poland, to access the medical records.

## Abbreviations

5-y-PSR: 5-Years prosthesis survival risk
ERN: European Reference Network
ESMO: European Society for Medical Oncology
EURACAN: European Reference Network for rare adult solid cancers
GMbH: Gesellschaft mit beschränkter Haftung-private German companies with limited liability
LR: Local recurrence
LSS: Limb-sparing surgery
MSTS: Musculoskeletal Tumor Society
NCCN: National Comprehensive Cancer Network
OS: Overall survival
PFS: Progression-free survival
R1: Non microscopically radical resection
